# Diabetes Mellitus in Patients Undergoing Mitral Transcatheter Edge-to-Edge Repair—A Decade Experience in 1000+ Patients

**DOI:** 10.3390/jcm12103502

**Published:** 2023-05-16

**Authors:** Michael Paukovitsch, Dominik Felbel, Matthias Groeger, Wolfgang Rottbauer, Sinisa Markovic, Marijana Tadic, Leonhard Moritz Schneider, Mirjam Keßler

**Affiliations:** University Heart Center Ulm, University Ulm, Albert-Einstein Allee 23, 89081 Ulm, Germany; michael.paukovitsch@uniklinik-ulm.de (M.P.); dominik.felbel@uniklinik-ulm.de (D.F.); matthias.groeger@uniklinik-ulm.de (M.G.); wolgang.rottbauer@uniklinik-ulm.de (W.R.); sinisa.markovic@uniklinik-ulm.de (S.M.); marijana.tadic@uniklinik-ulm.de (M.T.); leonhard-moritz.schneider@uniklinik-ulm.de (L.M.S.)

**Keywords:** edge-to-edge repair, mitral valve, diabetes

## Abstract

Background: Diabetes mellitus worsens outcomes in patients suffering from heart disease undergoing cardiac procedures. Objectives: To investigate the impact of diabetes in patients undergoing mitral transcatheter edge-to-edge repair (M-TEER). Methods: 1118 patients treated with M-TEER for functional (FMR) and degenerative (DMR) mitral regurgitation (MR) between 2010 and 2021 were analyzed using the combined endpoint of death/rehospitalization for heart failure (HFH). Results: Among diabetics (N = 306; 27.4%), comorbidities such as coronary artery disease (75.2% vs. 62.7%; *p* < 0.001) and progressed (stage III/IV) chronic kidney disease (79.5% vs. 72.6%; *p* = 0.018) were more frequent. The rate of FMR was higher in diabetics (71.9% vs. 64.5%; *p* < 0.001). The combined endpoint occurred more frequently in diabetics (40.2% vs. 35.6%; log-rank = 0.035). While no difference was observed in FMR patients (36.8% vs. 37.6%; log-rank *p* = 0.710), rates of the combined endpoint differed significantly between diabetics and non-diabetics in DMR patients (48.8% vs. 31.9%; log-rank *p* = 0.001) only. However, diabetes did neither predict the combined endpoint in the overall (OR: 0.97; 95% CI 0.65–1.45; *p* = 0.890) nor in the DMR cohort (OR: 0.73; 95% CI 0.35–1.51; *p* = 0.389). Among diabetics treated with M-TEER, troponin (OR: 2.32; 95% CI 1.3–3.7; *p* = 0.002) and estimated glomerular filtration rate (OR: 0.52; 95% CI 0.3–0.88; *p* = 0.018) independently predicted the combined endpoint. Conclusions: Diabetes is associated with adverse outcomes after M-TEER, particularly in DMR patients. However, diabetes does not predict the combined endpoint. In diabetics undergoing M-TEER, biochemical markers associated with organ function and damage independently predict the combined endpoint of death and rehospitalization.

## 1. Introduction

Mitral transcatheter edge-to-edge repair (M-TEER) has already been used for over a decade for the treatment of symptomatic mitral regurgitation (MR). In patients with prohibitive surgical but acceptable interventional risk, it provides a treatment option in addition to drug therapy. Studies have shown M-TEER to be both safe and effective in reducing MR in patients suffering from functional (FMR) or degenerative (DMR) mitral regurgitation [1,2,3]. Notably, among patients with heart failure, M-TEER reduces rehospitalization for heart failure and mortality [4]. Diabetes is a frequent risk factor predisposing patients to cardiac disease and is often observed in patients undergoing M-TEER [1,2,4,5,6,7]. Across the field of structural cardiac [8,9,10] and coronary interventions [11,12,13], ample evidence has been provided linking diabetes mellitus to adverse outcomes. In patients treated with M-TEER, a subgroup analysis from the COAPT (Cardiovascular Outcomes Assessment of the MitraClip Percutaneous Therapy for Heart Failure Patients with Functional Mitral Regurgitation) trial suggests worsened survival in diabetics with FMR [5], whereas others did not find a significant impact of diabetes on outcomes after M-TEER [14,15]. Given the conflicting evidence, we further investigated the impact of diabetes in >1000 patients undergoing M-TEER.

## 2. Materials and Methods

### 2.1. Study Population

For this single-center study, we retrospectively analyzed 1118 patients treated with M-TEER for FMR or DMR at our institution between 1 January 2010 and 31 December 2021. All patients undergoing M-TEER during the inclusion time frame were screened (N = 1171). Patients undergoing reintervention (N = 50) were excluded from this study. Reintervention rates were equally distributed among diabetics and non-diabetics (4.4% vs. 4.2%; *p* = 0.920). A patient was grouped into the diabetic group if a diagnosis of diabetes mellitus type II could be retrieved from current medical records at the time of the procedure. Patients with type I diabetes (N = 3) were excluded from this analysis. No further exclusion criteria existed.

Patients eligible for TEER suffered from chronic, symptomatic MR (grade III or IV) confirmed by transesophageal echocardiography (TEE) despite guideline-directed medical therapy (GDMT). Patients were evaluated by an interdisciplinary heart team and directed towards M-TEER by a joint decision based on guidelines [16,17,18] currently in place at the time of the procedure. All subjects were participants of the prospective MiTra ULM registry. All patients gave written informed consent for retrospective and prospective data collection. This research was approved by the local ethics committee.

The intervention was performed under general anesthesia, using echocardiographic guidance (TEE) and fluoroscopy. Precise details of M-TEER have been described elsewhere [2]. In brief, venous access is established via the groin and a guiding catheter is advanced towards the mitral valve after transseptal puncture from the right to the left atrium. One or multiple devices are positioned within the MV to achieve maximum MR reduction. The array of devices used in this study includes all commercially available edge-to-edge repair devices. For estimation of the glomerular filtration rate, the chronic kidney disease epidemiology collaboration (CKD-EPI) formula was used.

### 2.2. Patient Follow-Up

Standardized patient follow-up was completed by a routine clinical visit or telephone interview at 1, 3, 6 and 12 months and yearly thereafter. Patients not routinely seen in the outpatient department were followed up by a telephone interview carried out by trained study nurses.

### 2.3. Statistical Analysis

For statistical analysis, patients were grouped according to the presence of preexisting diabetes for the overall cohort and subgroups (FMR and DMR). The distribution of variables was analyzed using histograms and Q-Q plots. Continuous variables were expressed using mean and standard deviation. Categorical variables are shown as frequencies and percentages. Continuous variables were compared using Mann–Whitney Test or Student’s T-test depending on the distribution of variables. Outcomes were analyzed using the combined endpoint of all-cause death or rehospitalization for heart failure. Time-to-event analysis for the endpoint was performed using Kaplan–Maier curves and the log-rank test. The time-to-event data are shown as the median and its respective 95% confidence interval (CI). To identify parameters impacting the time-to-event patients were also grouped according to the endpoint. Univariate and multivariate Cox proportional hazards regression was used to quantify the impact of these parameters. Pearson and Spearman’s correlation coefficients as well as variance inflation factors (VIFs) were used to exclude correlation among variables before inclusion in multivariate models. Significant variables in univariate regression were included in the multivariate regression model using backwards conditional inclusion. For continuous variables, cut-off values were calculated using the Youden Index to maximize sensitivity and specificity. A *p*-value of <0.05 was considered significant for all statistical testing. Statistical analysis was carried out using SPSS, Version 29 (SPSS Statistics, IBM, Chicago, IL, USA).

## 3. Results

### 3.1. Baseline and Procedural Characteristics

Of 1118 patients, 306 (27.4%) were found to suffer from diabetes at the time of the procedure. FMR and mixed etiology were noted more often in diabetics (71.9% vs. 64.5%; *p* < 0.001). Table 1 depicts baseline patient characteristics for diabetics and non-diabetics. In Supplemental Table S1, this information is provided stratified according to etiology. Interventional risk as measured by the EuroSCORE II was significantly higher in diabetics (9.1 ± 7.5% vs. 7.9 ± 7.9%; *p* = 0.016). Symptom burden measured by New York Heart Association (NYHA) class was similar in diabetics and non-diabetics (*p* = 0.161), both in FMR (*p* = 0.635) and DMR (*p* = 0.104). In the overall cohort, diabetics were found to be significantly younger (76.0 ± 8.3 vs. 77.6 ± 9.1 years; *p* = 0.010). In subgroup analysis, this finding could be attributed to significantly younger FMR patients (74.7 ± 8.6 vs. 76.8 ± 9.4 years; *p* = 0.005), while no age difference was observed among DMR patients (79.0 ± 6.2 vs. 78.9 ± 8.2 years; *p* = 0.655).

Irrespective of etiology, diabetics had greater body mass index (BMI) compared to non-diabetics (27.7 ± 5.1 kg/m^2^ vs. 25.5 ± 4.6 kg/m^2^; *p* < 0.001). Comorbidities such as arterial hypertension (AHT: 87.9% vs. 76.8%; *p* < 0.001), coronary artery disease (CAD: 75.2% vs. 62.7%; *p* < 0.001) and progressed (stage III or IV) chronic kidney disease (CKD) (79.5% vs. 72.6%; *p* = 0.018) were more frequent among diabetics. Concerning CKD III/IV, in DMR patients this was found similar (86.0% vs. 67.2%; *p* < 0.001). Contrarily, in FMR patients, rates of CKD III/IV were equally frequent among diabetics and non-diabetics (77.0% vs. 75.5%; *p* = 0.678). Regarding heart failure medication, diabetics were prescribed more often with mineral corticoid receptor antagonists (MRA: 55.9% vs. 42.9%; *p* < 0.001), sodium-glucose-like transporter-2 inhibitors (SGLT-2i: 25.4% vs. 8.1%; *p* < 0.001) as well as angiotensin receptor neprilysin inhibitors (ARNI: 21.3% vs. 12.6%; *p* < 0.001). This was due to higher use of that medication in patients with FMR (see Supplemental Table S1), who had lower left-ventricular ejection fraction (LVEF: 36.0 ± 12.8% vs. 39.3 ± 15.2%; *p* = 0.008). LVEF did not differ between diabetics and non-diabetics with DMR (53.2 ± 13.1% vs. 54.8 ± 13.5%; *p* = 0.377).

Starting from similar preprocedural MR (3.7 ± 0.4 vs. 3.7 ± 0.5; *p* = 0.261), MR was reduced equally effective (1.3 ± 0.7 vs. 1.3 ± 0.7; *p* = 0.229) in diabetics and non-diabetics (further see Table 2). Hence, similar rates of optimal M-TEER results (MR ≤ I: 66.7% vs. 67.4%; *p* = 0.825) were noted in both etiologies (FMR: 65.5% vs. 70.8%; *p* = 0.785; DMR: 69.8% vs. 61.1%; *p* = 0.114).

Intra-hospital mortality was low in the overall cohort (2.6%) as well as in diabetics and non-diabetics (1.3% vs. 3.1%; *p* = 0.096). Furthermore, periprocedural rates of complications were comparable regarding adverse events, such as stroke (0.7% vs. 0.6%; Total: 0.6%; *p* = 0.938), sepsis (0.7% vs. 1.9%; Total: 1.5%; *p* = 0.178) and cardiogenic shock (1.0% vs. 2.3%; Total: 2.0%; *p* = 0.145). Moreover, 30-day mortality was also similar between diabetics and non-diabetics (2.9% vs. 4.2%; Total: 3.8%; *p* = 0.334).

### 3.2. Follow-Up and Outcome Analysis

**Figure 1:**
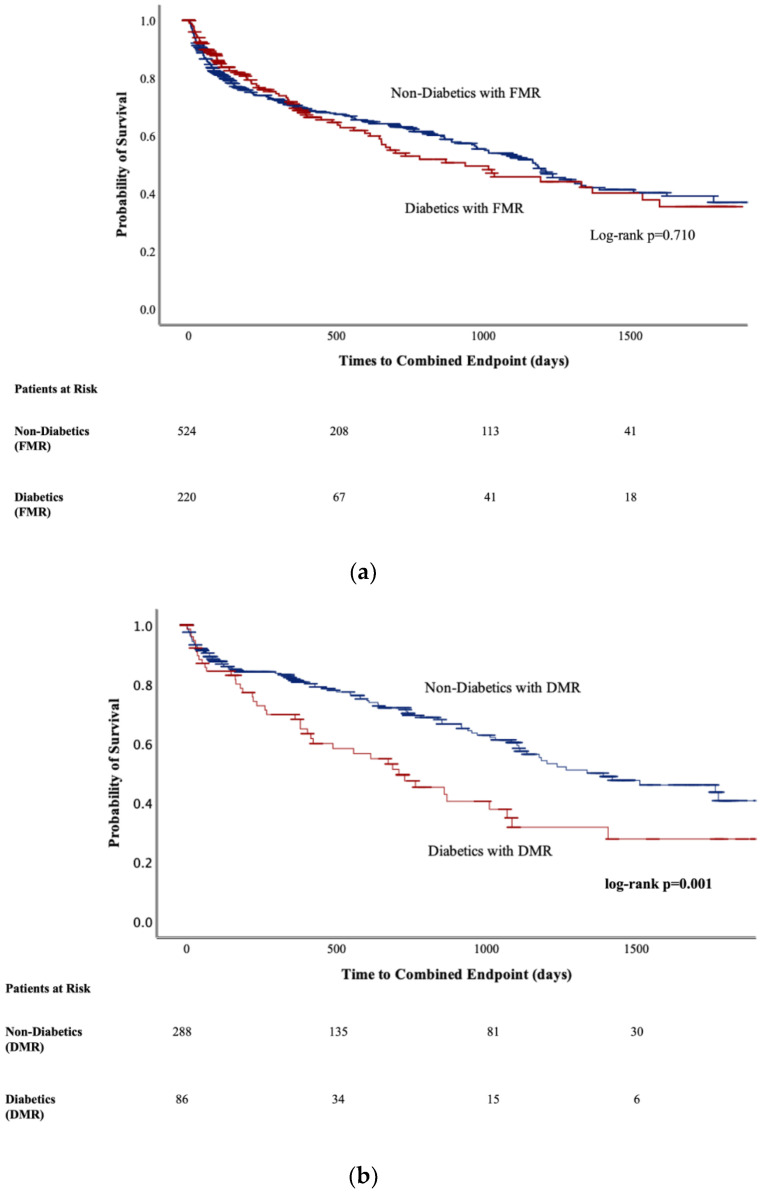
(**a**) Kaplan–Maier analysis of survival grouped according to non-diabetics and diabetics with functional mitral regurgitation (FMR). (**b**) Kaplan–Maier analysis of survival grouped according to non-diabetics and diabetics with degenerative mitral regurgitation (DMR).

The Graphic Abstract shows Kaplan–Meier time-to-event analysis for the combined endpoint of all-cause death and rehospitalization due to decompensated heart failure for the overall cohort. Stratified time-to-event analysis according to etiology is shown in Figure 1a,b. In the overall cohort, a significant difference (log-rank *p* = 0.035) regarding time-to-event (median (95% CI) in days) was observed between diabetics (784 (542–1026)) and non-diabetics (1202 (1085–1319)). About 40.2% of diabetics compared to 35.6% of non-diabetics reached the combined endpoint (*p* = 0.155). Stratified by etiology (Figure 1a,b), this observation was driven by DMR patients (708 (437–979) vs. 1336 (912–1759); log-rank *p* = 0.001) with event rates of 48.8% vs. 31.9% (*p* = 0.004) in diabetics and non-diabetics, respectively. In patients with FMR (Figure 1a), time-to-event data did not differ between diabetics and non-diabetics (939 (522–1356) vs. 1178 (1012–1343); log-rank *p* = 0.710; 36.8% vs. 37.6; *p* = 0.841). For further endpoint analysis, patients were stratified by the combined endpoint separately for the overall cohort (Supplemental Table S2) and patients with DMR (Supplemental Table S3). In multivariate Cox regression adjusted for etiology (see Table 3 and Table 4), diabetes did neither predict the combined endpoint in the overall cohort (OR: 0.97; 95% CI: 0.65–1.45; *p* = 0.890) nor in patients with DMR (OR: 0.73; 95% CI: 0.35–1.51; *p* = 0.389). However, female gender (0.68; 95% CI: 0.47–0.98; *p* = 0.038), NYHA class (1.59; 95% CI: 1.21–2.1; *p* = 0.004), troponin (1.92; 95% CI: 1.32–2.78; *p* < 0.001), NT-proBNP (1.73; 95% CI: 1.08–2.81; *p* = 0.022), hemoglobin level (0.67; 95% CI: 0.47–0.96; *p* = 0.033), periprocedural infection (2.36; 95% CI: 1.32–4.2; *p* = 0.004) and cardiogenic shock (3.2; 95% CI: 1.25–8.15; *p* = 0.015) independently predicted the combined endpoint in the overall cohort (see Table 3). In DMR patients, NYHA class (OR: 1.89; 95% CI: 1.12–3.0; *p* = 0.009), troponin (OR: 5.4; 95% CI: 2.3–12.6; *p* < 0.001) and statin use (OR: 0.47; 95% CI: 0.26–0.87; *p* = 0.016) were determined to predict the endpoint (see Table 4).

Since diabetes itself was neither found to predict the combined endpoint in the overall cohort nor in DMR patients, we investigated predictors of the combined endpoint among diabetics (N = 306) only (see Supplemental Table S4). Diabetics reaching the endpoint (40.2%) had higher average EuroSCORE II (11.1 ± 9.3 vs. 7.8 ± 6.4; *p* < 0.001), troponin (162.1 ± 671.1 µg/L vs. 47.7 ± 92.5µg/L; *p* = 0.036), NT-proBNP (7010.8 ± 7009.5 pg/mL vs. 4991.6 ± 5894.4 pg/mL; *p* = 0.016) and lower hemoglobin (11.6 ± 2.1 g/dL vs. 12.2 ± 1.9 g/dL; *p* = 0.015) as well as LVEF (37.5 ± 15.5% vs. 42.7 ± 14.4%; *p* = 0.005). Multivariate Cox regression (see Table 5) adjusted for etiology identified troponin (OR: 2.32; 95% CI: 1.3–3.7; *p* = 0.002) and eGFR (OR: 0.52; 95% CI: 0.3–0.88; *p* = 0.018) as independent predictors of the combined endpoint in diabetics.

## 4. Discussion

We investigated the impact of diabetes in >1000 patients undergoing M-TEER for treatment of DMR and FMR treated at a high-volume tertiary center. To the best of our knowledge, we were able to present data and analyze outcomes of diabetics in the largest real-world M-TEER cohort so far. The main findings of this study are as follows:

- Patients with diabetes can safely and effectively be treated with M-TEER.
- Diabetes is associated with adverse outcomes after M-TEER but does not independently predict these outcomes.
- In diabetics treated with M-TEER, well-established markers of advanced heart failure, troponin T and eGFR independently predict adverse outcomes.

Across studies reporting on M-TEER prevalence of diabetes ranges from 21.9 to 39.4% [1,2,4,5,6,7] making it a relevant comorbidity. Recently, an outcome analysis of diabetics [5] treated within the randomized-controlled (RCT) COAPT trial (Transcatheter Mitral-Valve Repair in Patients with Heart Failure) [4] was published. COAPT compared M-TEER to GDMT in FMR patients [4]. The investigators around Shahim et al. found a higher 2-year mortality risk in diabetics compared to non-diabetics. Yet, M-TEER consistently reduced 2-year mortality compared to guideline-directed medical therapy alone in diabetics and non-diabetics [5]. Unlike COAPT investigators, we did not see a difference in outcome between diabetics and non-diabetics in FMR patients. Moreover, the adverse outcomes associated with diabetes in our study were driven by DMR patients (see Figure 1). However, our patient population represents real-world data and does not seem to be strictly comparable to that from the randomized-controlled COAPT trial: Perioperative risk (assessed by STS Score) was similar between diabetics and non-diabetics with FMR in our study (5.5 ± 4.6% vs. 5.3 ± 6.2%; *p* = 0.667), while diabetics in the COAPT trial had higher perioperative risk (STS Score 5.1 ± 4.6% vs. 6.9 ± 6.5%; *p* < 0.001) compared to non-diabetics [5]. Renal disease was more frequent in their study in diabetics (65.1% vs. 51.9%; *p* = 0.002), while being balanced equally in our study (75.5% vs. 77.0%; *p* = 0.678) among FMR patients. Nevertheless, an overall greater comorbidity burden was seen among diabetics in our study, an observation also made by COAPT investigators [5] and others [14]. Apart from that, evidence regarding the outcome of diabetics after M-TEER is scarce: In a study by Hellhammer et al., diabetes independently predicted NT-proBNP non-response (≤30% decrease) at 6-month follow-up in a mixed cohort of 58 patients [19]. In another investigation of 79 patients with FMR by Paulus et al., diabetes predicted a lack of improvement in the six-minute walking test distance [20]. Nevertheless, these studies [15,20] neither unveil increased rehospitalization nor mortality rates in diabetics. Additionally, in a very recent study, Kirschfink et al. reported results from their real-world cohort of 340 patients with DMR and FMR: At 1-year follow-up, neither mortality nor rehospitalization differed between diabetics and non-diabetics [14].

In our study, Kaplan–Meier analysis for the combined endpoint of mortality/rehospitalization demonstrated a significant difference in time-to-event between diabetics and non-diabetics, which was driven by DMR patients. However, diabetes (OR: 0.97; 95% CI: 0.65–1.45; *p* = 0.890) itself was not found to independently predict the combined endpoint in multivariate Cox regression. After adjusting for MR etiology, female gender (OR: 0.67; 95% CI: 0.46–0.97; *p* = 0.038), NYHA class (OR: 1.59; 95% CI: 1.21–2.1; *p* = 0.004), troponin (OR: 1.92; 95% CI: 1.32–2.78; *p* < 0.001) and NT-proBNP (OR: 1.73; 95% CI: 1.08–2.81; *p* = 0.022) were identified as independent endpoint predictors instead. Troponin [21], NT-proBNP [22,23] and NYHA class [6,21,23,24,25,26] have previously been identified as independent predictors of mortality [6,22,23,24,25] or rehospitalization [21,26] after successful M-TEER. However, none of these studies reported an independent association of diabetes with adverse outcomes [6,21,22,23,24,25,26]. Despite these findings, the greater comorbidity burden associated with diabetes is conspicuous. Being a risk factor, diabetes predisposes to illness but harms with manifest disease. It is thus not surprising that we found troponin (OR: 2.32; 95% CI: 1.3–3.7; *p* = 0.002) and eGFR (OR: 0.52; 95% CI: 0.3–0.88; *p* = 0.018) to independently predict adverse outcomes in our study. Both denote organ damage or function and are well-established markers of disease in heart failure patients with diabetes: Troponin is a known independent predictor of adverse outcomes [27,28] and may help in finding diabetics at risk for cardiovascular disease [29]. The relationship between renal function and adverse outcomes in diabetics is also well-established [30,31].

With regard to the prevalence of comorbidities in patients with FMR and DMR, some interesting observations became evident: Hyperlipidemia (HLP) was significantly more prevalent among diabetics with FMR (*p* < 0.01), whereas the rate of current smokers was significantly higher in DMR patients (*p* = 0.019). These effects were not observed vice-versa with similar rates of HLP (*p* = 0.734) and smoking status (0.182) in DMR and FMR patients, respectively. These findings are interesting; however, unlikely to have any effect on the outcome as neither variable was found to predict outcome in the overall cohort nor in diabetics.

In our study, rates of periprocedural adverse events were low in the overall cohort with no difference in diabetics and non-diabetics. Furthermore, the results of M-TEER did not vary either. Consequently, our study is in line with others [5,14], confirming the safety and effectiveness of M-TEER in diabetics.

## 5. Limitations

We presented results from an observational retrospective single-center investigation with all limitations inherent in such a study. Our study’s population reflects treatment strategy and patient selection in a German high-volume tertiary center over the course of more than 10 years in >1000 patients. We demonstrated that these patients differ from those treated in a large RCT (COAPT), which operates on tighter inclusion and exclusion criteria. Moreover, guidelines as well as patient selection for M-TEER have changed in recent years and any effects thereof, which might act as confounders, cannot be ruled out. Data on diabetes onset and glycemic control over time were not available retrospectively. Our results could therefore not be adjusted for these factors and a confounding effect cannot be ruled out. Moreover, poor glycemic control was associated with worse outcomes in previous studies in patients suffering from diabetes [32] as well as in diabetics with heart failure [33].

## 6. Conclusions

Diabetes is associated with adverse outcomes after M-TEER, particularly in DMR patients. However, biochemical markers associated with organ function (eGFR) and damage (Troponin) predict the combined endpoint of death and rehospitalization, whereas diabetes mellitus itself does not.

## Figures and Tables

**Table 1:** Baseline patient characteristics and echocardiography.

Parameter	No Diabetes (N = 812)	Diabetes (N = 306)	Total (N = 1118)	*p*
Age	77.6 ± 9.1	76.0 ± 8.3	77.1 ± 8.6	**0.010**
BMI (kg/m^2^) N = 1113	25.5 ± 4.6	27.7 ± 5.1	26.0 ± 4.8	**<0.001**
Sex, N (female) (%)	344 (42.4)	124 (40.5)	468 (41.9)	0.587
Arterial hypertension, N (%)	624 (76.8)	269 (87.9)	893 (79.9)	**<0.001**
CAD, N (%)	509 (62.7)	230 (75.2)	739 (66.1)	**<0.001**
Prior MI	167 (20.6)	108 (35.3)	275 (24.6)	**<0.001**
Hyperlipidemia, N (%)	456 (56.2)	196 (64.1)	652 (58.3)	**0.02**
Pulmonary hypertension, N (%)	321 (39.5)	109 (35.6)	430 (38.5)	0.231
COPD, N (%)	82 (10.1)	40 (13.1)	122 (10.9)	0.163
Smoker (current/former), N (%)	155 (19.1)	79 (25.8)	234 (20.9)	**0.014**
Family disposition, N (%)	113 (13.9)	50 (16.3)	163 (14.6)	0.306
AF, N (%)	542 (66.7)	186 (60.8)	728 (65.1)	0.062
LBBB, N (%) (N = 1111)	140 (17.2)	70 (22.9)	210 (18.8)	**0.032**
CRT-D/P, N (%)	67 (8.3)	28 (9.2)	95 (8.5)	0.631
DMR, N (%)	288 (35.5)	86 (28.1)	374 (33.5)	**<0.001**
FMR, N (%)	371 (45.7)	173 (56.5)	544 (48.7)	
Mixed etiology, N (%)	153 (18.8)	47 (15.4)	200 (17.9)	
FMR/mixed, N (%)	524 (64.5)	220 (71.9)	744 (71.9)	**<0.001**
DCM, N (%)	164 (20.2)	53 (17.3)	217 (19.4)	0.228
NYHA II, N (%)	126 (15.5)	39 (12.7)	165 (14.8)	0.161
NYHA III, N (%)	484 (59.6)	175 (57.2)	659 (58.9)	
NYHA IV, N (%)	202 (24.9)	92 (30.1)	294 (26.3)	
EuroSCORE II	7.9 ± 7.9	9.1 ± 7.5	8.2 ± 7.9	**0.016**
STS risk of mortality score	5.2 ± 6.5	5.8 ± 5.3	5.3 ± 6.2	0.139
Troponin T pre (µg/L) N = 969	103.0 ± 682.9	91.1 ± 426.0	100.0 ± 625.1	0.809
NT-pro BNP pre (pg/mL) N = 933	5028.1 ± 6017.2	5712.7 ± 6375.6	5213.0 ± 6120	0.129
Hemoglobin level (g/dL)	12.5 ± 1.9	12.0 ± 2.0	12.3 ± 1.9	**<0.001**
eGFR (mL/min)	50.0 ± 20.1	45.7 ± 19.4	48.8 ± 20.0	**0.001**
CKD III/IV	583 (72.6)	241 (79.5)	824 (74.5)	**0.018**
**Medication**				
BB, N (%)	682 (84.0)	270 (88.2)	952 (85.2)	0.075
ACEI, N (%)	325 (40.0)	114 (37.3)	439 (39.3)	0.398
ARB, N (%)	235 (28.9)	85 (27.8)	320 (28.6)	0.701
ARNI, N (%)	79 (12.6)	49 (21.3)	128 (14.9)	**<0.001**
MRA, N (%)	348 (42.9)	171 (55.9)	519 (46.4)	**<0.001**
SGLT-2 inhibitors, N (%) N = 476	28 (8.1)	33 (25.4)	61 (12.8)	**<0.001**
Loop diuretics, N (%)	612 (75.4)	255 (83.3)	867 (77.5)	**0.004**
Statins, N (%)	496 (61.1)	234 (76.5)	730 (65.3)	**<0.001**
ASS, N (%)	272 (33.5)	138 (45.1)	410 (36.7)	**<0.001**
NOAC, N (%)	408 (50.2)	138 (45.1)	546 (48.8)	0.125
P2Y12 inhibitor, N (%)	173 (21.3)	91 (29.7)	264 (23.6)	**0.003**
**Echocardiography**				
LVEF (%)	44.4 ± 16.3	40.7 ± 15.0	43.4 ± 16.1	**<0.001**
LVEDd (mm) N = 807	59.3 ± 11.6	61.0 ± 11.0	59.8 ± 11.4	0.069
LVESd (mm) N = 765	44.4 ± 13.8	47.0 ± 13.0	45.1 ± 13.6	**0.021**
IVSd (mm) N = 830	10.8 ± 2.3	11.1 ± 7.1	10.9 ± 4.2	0.372
LA diameter (mm) N = 851	55.1 ± 9.5	54.0 ± 8.0	54.8 ± 9.2	0.159
mPG pre (mmHg)	1.8 ± 1.3	1.9 ± 1.3	1.8 ± 1.3	0.027
mPG post (mmHg)	3.5 ± 1.7	3.7 ± 1.7	3.6 ± 1.7	0.285
Grade of TR	1.8 ± 1.0	1.6 ± 1.0	1.8 ± 1.0	**<0.001**
Severe TR (Grade III)	258 (33.0)	83 (27.1)	351 (31.4)	0.059
sPAP (mmHg)	43.6 ± 25.7	40.3 ± 27.0	42.7 ± 26.1	0.060

Values are shown as frequencies (N) and percentages (%), mean ± standard deviation (SD). Significant *p*-values are in bold letters. Abbreviations: BMI = body mass index (kg/m^2^); CAD = coronary artery disease; MI = myocardial infarction; COPD = chronic obstructive pulmonary disease; AF = atrial fibrillation; LBBB = left bundle branch block; CRT = cardiac resynchronization therapy; DCM = dilatative cardiomyopathy; DMR = degenerative mitral regurgitation; FMR = functional mitral regurgitation; NYHA = New York Heart Association; STS = Society of Thoracic Surgeons; NT-proBNP = N-terminal pro hormone brain natriuretic peptide; eGFR = estimated glomerular filtration rate; BB = beta blocker; ACEI = angiotensin-converting enzyme inhibitor; ARB = AT receptor blocker; ARNI = angiotensin–neprilysin inhibitor; MRA = mineralocorticoid receptor antagonist; SGLT-2 = sodium-glucose cotransporter-2; ASS = acetylic salicylic acid; NOAC = novel oral anticoagulant; P2Y12 inhibitor = adenosine diphosphate receptor antagonists; MR = mitral regurgitation; MV = mitral valve; LVEF = left-ventricular ejection fraction; LVEDd = left-ventricular end-diastolic diameter; LVESd = left-ventricular end-systolic diameter; LA = left atrium; IVSd = septum diameter; sPAP = systolic pulmonary artery pressure; TR = tricuspid regurgitation; PG = pressure gradient.

**Table 2:** Procedural outcomes.

Parameter	No Diabetes (N = 812)	Diabetes (N = 306)	Total (N = 1118)	*p*
Grade of MR preprocedural (I–IV)	3.7 ± 0.5	3.7 ± 0.4	3.7 ± 0.5	0.261
III	251 (30.9)	84 (27.5)	335 (30.0)	0.260
IV	561 (69.1)	222 (72.5)	783 (70.0)	
Grade of MR postprocedural	1.3 ± 0.7	1.3 ± 0.7	1.3 ± 0.7	0.229
Grade ≤ I	547 (67.4)	204 (66.7)	751 (67.2)	0.825
tabProcedure time (s) N = 862	5175.9 ± 3307.3	5415.5 ± 2477.1	5239.8 ± 3105.7	0.546
Fluoroscopy time (s) N = 862	1677.5 ± 871.1	1647.1 ± 869.8	1669.5 ± 869.8	0.651
Number of implanted devices	1.4 ± 0.6	1.4 ± 0.5	1.4 ± 0.6	0.351

Values are shown as frequencies (N) and percentages (%), mean ± standard deviation (SD). Abbreviations: MR = mitral regurgitation.

**Table 3:** Univariate and multivariate Cox regression for predictors of death/rehospitalization in the overall cohort (N = 1118).

Univariate				Multivariate		
Parameter	OR	95% CI	*p*	OR	95% CI	*p*
Female	0.76	0.62–0.94	**0.009**	0.68	0.47–0.98	**0.038**
CAD	1.15	0.93–1.43	0.190			
Prior MI	1.58	1.28–1.9	**<0.001**			
COPD	1.37	1.04–1.8	**0.034**			
CRT-D/P	1.31	0.96–1.79	0.099			
NYHA class	1.7	1.46–1.97	**<0.001**	1.59	1.21–2.1	**0.004**
Troponin (µg/L)	2.76	2.2–3.47	**<0.001**	1.92	1.32–2.78	**<0.001**
NT-proBNP (pg/mL)	2.7	2.04–3.57	**<0.001**	1.73	1.08–2.81	**0.022**
LA diameter (mm)	1.33	1.06–1.67	**0.013**			
sPAP (mmHg)	1.52	1.15–1.99	**0.003**			
Hb (g/dL)	0.55	0.45–0.67	**<0.001**	0.67	0.47–0.96	**0.033**
eGFR (mL/min)	0.53	0.44–0.65	**<0.001**			
LVEF (%)	0.59	0.48–0.72	**<0.001**			
Loop diuretics	1.74	1.34–2.25	**<0.001**			
NOAC	0.96	0.79–1.17	0.682			
CKD III/IV	1.83	1.42–2.36	**0.001**			
Severe TR	1.3	1.06–1.6	**0.015**			
Grade of TR	1.19	1.07–1.3	**0.006**			
Grade of MR preprocedural	1.36	1.07–1.7	**0.012**			
Grade of MR (residual)	0.89	0.73–1.08	0.229			
No. of devices	0.95	0.83–1.08	0.441			
Cardiogenic shock	5.4	3.33–8.81	**<0.001**	3.2	1.25–8.15	**0.015**
Infection	2.92	2.1–4.0	**<0.001**	2.36	1.32–4.2	**0.004**
Pneumonia	3.63	2.45–5.37	**<0.001**			
Sepsis	10.46	6.3–17.4	**<0.001**			
Diabetes	1.25	1.02–1.55	**0.036**	0.97	0.65–1.45	0.890
Etiology FMR/mixed				0.91	0.63–1.33	0.672

Variables are shown as odds ratios (Ors) and 95% confidence intervals (Cis). Significant *p*-values are in bold letters. Using backwards conditional inclusion, the following variables were included in multivariate Cox regression (adjusted for etiology): LVEF, CAD, troponin, Nt-proBNP, LA diameter, sPAP, Hb, eGFR, Sex, NYHA class, loop diuretics, COPD, severe TR, infection and FMR = functional mitral regurgitation. Prior MI, pneumonia and sepsis were not included for due to autocorrelation with CAD and infection, respectively (r > 0.4). Variables remaining significant in multivariate regression are shown in the respective table section (right).

**Table 4:** Univariate and multivariate Cox regression for predictors of death/rehospitalization in patients with DMR (N = 374).

Univariate				Multivariate		
Parameter	OR	95% CI	*p*	OR	95% CI	*p*
Female	0.76	0.54–1.08	0.124			
Diabetes	1.8	1.25–2.6	**0.002**			
CAD	1.22	0.85–1.74	0.285			
NYHA class	1.64	1.25–2.14	**<0.001**	1.89	1.18–3.0	**0.009**
Troponin (µg/L)	3.28	2.04–5.28	**<0.001**	5.4	2.3–12.3	**<0.001**
NT-proBNP (pg/mL)	2.68	1.76–4.1	**<0.001**			
LA diameter	1.02	1.001–1.04	**0.043**			
Hb (g/dL)	0.47	0.33–0.66	**<0.001**			
eGFR (mL/min)	0.4	0.28–0.56	**<0.001**			
Loop diuretics	2.1	1.37–3.29	**<0.001**			
CKD III/IV	1.9	1.22–2.96	**0.004**			
Statins	0.69	0.49–0.96	**0.030**	0.47	0.26–0.87	**0.016**
ARNI	2.86	1.43–5.72	**0.010**			
Grade of MR preprocedural	2.04	1.07–3.9	**0.030**			
Grade of MR (residual)	0.83	0.59–1.17	0.298			
mPG post (mmHg)	1.14	1.01–1.29	**0.045**			
Cardiogenic shock	7.45	3.45–16.1	**<0.001**			
Sepsis	5.1	2.08–12.56	**<0.001**			

Variables are shown as odds ratios (ORs) and 95% confidence intervals (CIs). Significant *p*-values are in bold letters. Using backwards conditional inclusion, the following variables were included in multivariate Cox regression: diabetes, NYHA class, troponin, Nt-proBNP, LA diameter, Hb, eGFR, loop diuretics, statins, ARNI, mPG post and sepsis. Cardiogenic shock was not included due to multicollinearity (r > 0.4) with sepsis. Only variables remaining significant in multivariate regression are shown in the respective table section (right).

**Table 5:** Univariate and multivariate Cox regression for predictors of death/rehospitalization in patients with diabetes (N = 306).

Univariate				Multivariate		
Parameter	OR	95% CI	*p*	OR	95% CI	*p*
Troponin (µg/L)	2.20	1.48–3.89	**<0.001**	2.32	1.3–3.7	**0.002**
NT-proBNP (pg/mL)	2.2	1.45–3.34	**<0.001**			
Hb (g/dL)	0.7	0.47–1.04	0.074			
eGFR (mL/min)	0.98	0.98–0.99	**0.001**	0.52	0.30–0.88	**0.018**
CKD III/IV	1.81	1.1–3.02	**0.024**			
SGLTI-2	0.59	0.24–1.4	0.207			
LVEF (%)	0.57	0.39–0.85	**0.005**			
LVEDD (mm)	1.02	0.99–1.04	0.062			
LVESD (mm)	1.03	1.01–1.04	**0.001**			
Grade of MR preprocedural	1.16	0.75–1.79	0.501			
Infection	2.4	1.26–4.6	**0.019**	2.34	0.98–5.5	0.057
Pneumonia	4.13	1.8–9.47	**<0.001**			
Etiology (FMR and mixed)	0.82	0.56–1.19	0.290	0.68	0.42–1.1	0.139

Variables are shown as odds ratios (ORs) and 95% confidence intervals (CIs). Significant *p*-values are in bold letters. Using backwards conditional inclusion, the following variables were included in multivariate Cox regression (adjusted for etiology): troponin, Nt-proBNP, infection, LVEF and eGFR. Pneumonia, LVESD/LVESD and CKD were not included due to multicollinearity (r > 0.4) with infection LVEF and eGFR, respectively. Only variables remaining significant in multivariate regression are shown in the respective table section (right).

## Data Availability

All relevant data are included within the manuscript or its Supplemental Information.

## Supplementary Materials

The following supporting information can be downloaded at https://www.mdpi.com/article/10.3390/jcm12103502/s1, a supplementary file containing additional tables is attached to this manuscript. Table S1. Baseline patient characteristics, echocardiography and procedural outcomes separated by etiology. Table S2. Patients (overall cohort) grouped according to the endpoint of death/rehospitalization for heart failure. Table S3. Patients (DMR only) grouped according to the endpoint of death/rehospitalization for heart failure. Table S4. Diabetics grouped according to the endpoint of death/rehospitalization for heart failure.

## References

[1] Glower D.D., Kar S., Trento A., Lim D.S., Bajwa T., Quesada R., Whitlow P.L., Rinaldi M.J., Grayburn P., Mack M.J. (2014). Percutaneous mitral valve repair for mitral regurgitation in high-risk patients: Results of the EVEREST II study. J. Am. Coll. Cardiol..

[2] Feldman T., Kar S., Rinaldi M., Fail P., Hermiller J., Smalling R., Whitlow P.L., Gray W., Low R., Herrmann H.C. (2009). Percutaneous Mitral Repair with the MitraClip System. Safety and Midterm Durability in the Initial EVEREST (Endovascular Valve Edge-to-Edge REpair Study) Cohort. J. Am. Coll. Cardiol..

[3] Feldman T., Foster E., Glower D.D., Kar S., Rinaldi M.J., Fail P.S., Smalling R.W., Siegel R., Rose G.A., Engeron E. (2011). Percutaneous Repair or Surgery for Mitral Regurgitation. N. Engl. J. Med..

[4] Stone G.W., Lindenfeld J., Abraham W.T., Kar S., Lim D.S., Mishell J.M., Whisenant B., Grayburn P.A., Rinaldi M., Kapadia S.R. (2018). Transcatheter mitral-valve repair in patients with heart failure. N. Engl. J. Med..

[5] Shahim B., Ben-Yehuda O., Chen S., Redfors B., Madhavan M.V., Kar S., Lim D.S., Asch F.M., Weissman N.J., Cohen D.J. (2021). Impact of Diabetes on Outcomes After Transcatheter Mitral Valve Repair in Heart Failure: COAPT Trial. JACC Heart Fail..

[6] Kalbacher D., Schäfer U., Bardeleben R.S.V., Eggebrecht H., Sievert H., Nickenig G., Butter C., May A.E., Bekeredjian R., Ouarrak T. (2019). Long-term outcome, survival and predictors of mortality after MitraClip therapy: Results from the German Transcatheter Mitral Valve Interventions (TRAMI) registry. Int. J. Cardiol..

[7] Maisano F., Franzen O., Baldus S., Schäfer U., Hausleiter J., Butter C., Ussia G.P., Sievert H., Richardt G., Widder J.D. (2013). Percutaneous mitral valve interventions in the real world: Early and 1-year results from the ACCESS-EU, A prospective, multicenter, nonrandomized post-approval study of the Mitraclip therapy in Europe. J. Am. Coll. Cardiol..

[8] Ando T., Takagi H., Briasoulis A., Umemoto T. (2017). Does diabetes mellitus impact prognosis after transcatheter aortic valve implantation? Insights from a meta-analysis. J. Cardiol..

[9] Matsumoto S., Ohno Y., Miyamoto J., Ikari Y., Tada N., Naganuma T., Yamawaki M., Yamanaka F., Shirai S., Mizutani K. (2021). Impact of diabetes mellitus on outcome after transcatheter aortic valve replacement: Identifying high-risk diabetic population from the OCEAN-TAVI registry. Catheter. Cardiovasc. Interv..

[10] Conrotto F., D’Ascenzo F., Giordana F., Salizzoni S., Tamburino C., Tarantini G., Presbitero P., Barbanti M., Gasparetto V., Mennuni M. (2014). Impact of diabetes mellitus on early and midterm outcomes after transcatheter aortic valve implantation (from a multicenter registry). Am. J. Cardiol..

[11] Mathew V., Gersh B.J., Williams B.A., Laskey W.K., Willerson J.T., Tilbury R.T., Davis B.R., Holmes J.D.R. (2004). Outcomes in Patients with Diabetes Mellitus Undergoing Percutaneous Coronary Intervention in the Current Era: A Report from the Prevention of REStenosis with Tranilast and its Outcomes (PRESTO) Trial. Circulation.

[12] Verma S., Farkouh M.E., Yanagawa B., Fitchett D.H., Ahsan M.R., Ruel M., Sud S., Gupta M., Singh S., Gupta N. (2013). Comparison of coronary artery bypass surgery and percutaneous coronary intervention in patients with diabetes: A meta-analysis of randomised controlled trials. Lancet Diabetes Endocrinol..

[13] Chichareon P., Modolo R., Kogame N., Takahashi K., Chang C.-C., Tomaniak M., Botelho R., Eeckhout E., Hofma S., Trendafilova-Lazarova D. (2020). Association of diabetes with outcomes in patients undergoing contemporary percutaneous coronary intervention: Pre-specified subgroup analysis from the randomized GLOBAL LEADERS study. Atherosclerosis.

[14] Kirschfink A., Alachkar M.N., Alnaimi A., Vogt F., Schroeder J., Lehrke M., Frick M., Reith S., Marx N., Almalla M. (2022). Outcome of transcatheter edge-to-edge mitral valve repair in patients with diabetes mellitus: Results from a real-world cohort. PLoS ONE.

[15] Hellhammer K., Zeus T., Balzer J., Van Hall S., Rammos C., Wagstaff R., Kelm M., Rassaf T. (2014). Safety and efficacy of percutaneous mitral valve repair using the MitraClip^®^ system in patients with diabetes mellitus. PLoS ONE.

[16] Baumgartner H., Falk V., Bax J.J., De Bonis M., Hamm C., Holm P.J., Iung B., Lancellotti P., Lansac E., Rodriguez Muñoz D. (2017). 2017 ESC/EACTS Guidelines for the management of valvular heart disease. Eur. Heart J..

[17] Vahanian A., Beyersdorf F., Praz F., Milojevic M., Baldus S., Bauersachs J., Capodanno D., Conradi L., De Bonis M., De Paulis R. (2021). 2021 ESC/EACTS Guidelines for the management of valvular heart disease. Eur. Heart J..

[18] Vahanian A., Alfieri O., Andreotti F., Antunes M.J., Barón-Esquivias G., Baumgartner H., Borger M.A., Carrel T.P., De Bonis M., Evangelista A. (2012). Guidelines on the management of valvular heart disease (version 2012). Eur. Heart J..

[19] Kaneko H., Neuss M., Weissenborn J., Butter C. (2017). Role of right ventricular dysfunction and diabetes mellitus in n-terminal pro-b-type natriuretic peptide response of patients with severe mitral regurgitation and heart failure after mitraclip. Int. Heart J..

[20] Paulus M.G., Meindl C., Böhm L., Holzapfel M., Hamerle M., Schach C., Maier L.S., Debl K., Unsöld B., Birner C. (2020). Predictors of functional improvement in the short term after MitraClip implantation in patients with secondary mitral regurgitation. PLoS ONE.

[21] Keßler M., Seeger J., Muche R., Wöhrle J., Rottbauer W., Markovic S. (2019). Predictors of rehospitalization after percutaneous edge-to-edge mitral valve repair by MitraClip implantation. Eur. J. Heart Fail..

[22] Kitamura M., Kaneko H., Schlüter M., Schewel D., Schmidt T., Alessandrini H., Kreidel F., Neuss M., Butter C., Kuck K.-H. (2019). Predictors of mortality in ischaemic versus non-ischaemic functional mitral regurgitation after successful transcatheter mitral valve repair using MitraClip: Results from two high-volume centres. Clin. Res. Cardiol..

[23] Triantafyllis A.S., Kortlandt F., Bakker A.L., Swaans M.J., Eefting F.D., van der Heyden J.A., Post M.C., Rensing B.W. (2016). Long-term survival and preprocedural predictors of mortality in high surgical risk patients undergoing percutaneous mitral valve repair. Catheter. Cardiovasc. Interv..

[24] Capodanno D., Adamo M., Barbanti M., Giannini C., Laudisa M.L., Cannata S., Curello S., Immè S., Maffeo D., Bedogni F. (2015). Predictors of clinical outcomes after edge-to-edge percutaneous mitral valve repair. Am. Heart J..

[25] Puls M., Lubos E., Boekstegers P., von Bardeleben R.S., Ouarrak T., Butter C., Zuern C.S., Bekeredjian R., Sievert H., Nickenig G. (2016). One-year outcomes and predictors of mortality after MitraClip therapy in contemporary clinical practice: Results from the German transcatheter mitral valve interventions registry. Eur. Heart J..

[26] Puls M., Tichelbäcker T., Bleckmann A., Hünlich M., von der Ehe K., Beuthner B.E., Rüter K., Beißbarth T., Seipelt R., Schöndube F. (2014). Failure of acute procedural success predicts adverse outcome after percutaneous edge-to-edge mitral valve repair with MitraClip. Eurointervention.

[27] Everett B.M., Brooks M.M., Vlachos H.E.A., Chaitman B.R., Frye R.L., Bhatt D.L. (2015). Troponin and Cardiac Events in Stable Ischemic Heart Disease and Diabetes. N. Engl. J. Med..

[28] Lepojärvi E.S., Piira O.-P., Kiviniemi A.M., Miettinen J.A., Kenttä T., Ukkola O., Tulppo M.P., Huikuri H.V., Junttila M.J. (2016). Usefulness of Highly Sensitive Troponin as a Predictor of Short-Term Outcome in Patients with Diabetes Mellitus and Stable Coronary Artery Disease (from the ARTEMIS Study). Am. J. Cardiol..

[29] Gori M., Gupta D.K., Claggett B., Selvin E., Folsom A.R., Matsushita K., Bello N.A., Cheng S., Shah A., Skali H. (2016). Natriuretic peptide and high-sensitivity troponin for cardiovascular risk prediction in diabetes: The Atherosclerosis Risk in Communities (ARIC) study. Diabetes Care.

[30] Fox C.S., Matsushita K., Woodward M., Bilo H.J., Chalmers J., Heerspink H.J.L., Lee B.J., Perkins R.M., Rossing P., Sairenchi T. (2012). Associations of kidney disease measures with mortality and end-stage renal disease in individuals with and without diabetes: A meta-analysis. Lancet.

[31] Caplan E.O., Sheer R., Schmedt N., Evers T., Cockrell M., Tindal M., Pasquale M.K., Kovesdy C.P. (2021). Glomerular filtration rate change and outcomes in type 2 diabetes. Am. J. Manag. Care.

[32] Hayward R.A., Reaven P.D., Wiitala W.L., Bahn G.D., Reda D.J., Ge L., McCarren M., Duckworth W.C., Emanuele N.V. (2015). Follow-up of Glycemic Control and Cardiovascular Outcomes in Type 2 Diabetes. N. Engl. J. Med..

[33] Paolillo S., Salvioni E., Filardi P.P., Bonomi A., Sinagra G., Gentile P., Gargiulo P., Scoccia A., Cosentino N., Gugliandolo P. (2020). Long-term prognostic role of diabetes mellitus and glycemic control in heart failure patients with reduced ejection fraction: Insights from the MECKI Score database. Int. J. Cardiol..

