# Supplementary material for: Diabetes Mellitus in Patients Undergoing Mitral Transcatheter Edge-to-Edge Repair—A Decade Experience in 1000+ Patients
Source: J Clin Med. 2023 May 16;12(10):3502. doi: 10.3390/jcm12103502 (PMC10219195; doi:10.3390/jcm12103502)
Supplement: Supplementary file 1 [file jcm-12-03502-s001.zip › jcm-2376166-supplementary.pdf]

SUPPLEMENTAL TABLES

Supplemental Table S1. Baseline patient characteristics, echocardiography and procedural outcomes separated by etiology

	FMR				DMR			
**Parameter**	**No Diabetes (N = 524)**	**Diabetes (N = 220)**	**Total (N = 744)**	**p**	**No Diabetes (N = 288)**	**Diabetes (N = 86)**	**Total (N = 374)**	**p**
Age	76.8 ± 9.4	74.7 ± 8.6	76.2 ± 9.2	**0.005**	78.9 ± 8.2	79.0 ± 6.2	79.0 ± 7.8	0.655
BMI (kg/m^2) N = 1113	25.5 ± 4.6	27.6 ± 5.2	26.1 ± 4.5	**<0.001**	25.4 ± 4.5	27.9 ± 5.0	25.9 ± 4.7	**<0.001**
Sex, N (female) (%)	208 (39.7)	85 (38.6)	293 (39.4)	0.787	136 (47.2)	39 (45.3)	174 (46.8)	0.760
Arterial hypertension, N (%)	402 (76.7)	192 (87.3)	594 (79.8)	**0.001**	222 (77.1)	77 (89.5)	299 (79.9)	**0.011**
CAD, N (%)	350 (66.8)	171 (77.7)	521 (70.0)	**0.003**	159 (55.2)	59 (68.6)	218 (58.3)	**0.027**
Prior MI	133 (25.4)	90 (40.9)	223 (30.0)	**<0.001**	34 (11.8)	18 (20.9)	52 (13.9)	**0.049**
Hyperlipidemia, N (%)	306 (58.4)	153 (69.5)	459 (61.7)	**<0.01**	150 (52.1)	43 (50.0)	193 (51.6)	0.734
Pulmonary hypertension, N (%)	204 (38.9)	79 (35.9)	283 (38.0)	0.438	117 (40.6)	30 (34.9)	147 (39.3)	0.339
COPD, N (%)	57 (10.9)	27 (12.3)	84 (11.3)	0.612	25 (8.7)	13 (15.1)	38 (10.2)	0.083
Smoker (current/former), N (%)	110 (21.0)	56 (25.5)	166 (22.3)	0.182	45 (15.6)	23 (26.7)	68 (18.2)	**0.019**
Family disposition, N (%)	80 (15.3)	41 (18.6)	121 (16.3)	0.256	33 (11.5)	9 (10.5)	42 (11.2)	0.798
AF, N (%)	357 (68.1)	130 (59.1)	487 (65.5)	0.018	185 (54.2)	56 (65.1)	241 (64.4)	0.881
LBBB, N (%) (N = 1111)	113 (21.6)	58 (26.4)	171 (23.0)	0.156	27 (9.4)	12 (14.0)	39 (10.4)	0.223
CRT-D/P, N (%)	61 (11.6)	26 (11.8)	87 (11.7)	0.945	6 (2.1)	2 (2.3)	9 (2.1)	0.892
DCM, N (%)	139 (26.5)	48 (21.8)	187 (25.1)	0.177	25 (8.7)	5 (5.8)	30 (8.0)	0.500
NYHA II, N (%)	78 (14.9)	31 (14.1)	109 (14.7)	0.635	48 (16.7)	8 (9.3)	56 (15.0)	0.104
NYHA III, N (%)	309 (59.0)	124 (56.4)	433 (58.2)		175 (60.8)	51 (59.3)	226 (60.4)	

NYHA IV, N (%)	137 (26.1)	65 (29.5)	202 (27.2)		65 (22.6)	27 (31.4)	92 (24.6)	
EuroSCORE II	8.3 ± 8.0	9.3 ± 7.5	8.6 ± 7.9	0.141	7.0 ± 7.6	8.8 ± 8.7	7.4 ± 7.9	0.061
STS risk of mortality score	5.3 ± 6.2	5.5 ± 4.6	5.3 ± 5.8	0.667	4.9 ± 7.0	6.5 ± 6.7	5.3 ± 7.0	0.065
Troponin T pre (µg/L) N = 969	137.4 ± 835.3	112.8 ± 500.9	130.5 ± 756.5	0.708	34.7 ± 37.0	38.7 ± 30.7	35.6 ± 35.6	0.413
NT-pro BNP pre (pg/mL) N = 933	5768.9 ± 6318.1	6138.0 ± 6757.7	5876.2 ± 6445.6	0.515	3612.8 ± 5117.8	4584.7 ± 5102.5	3834.2 ± 5122.1	0.166
Hemoglobin level (g/dL)	12.4 ± 2.0	12.1 ± 2.0	12.3 ± 2.0	**0.023**	12.6 ± 1.7	11.7 ± 2.1	12.4 ± 1.9	**<0.001**
eGFR (mL/min)	48.0 ± 19.4	46.1 ± 20.4	47.4 ± 19.7	0.222	53.6 ± 20.8	44.6 ± 16.8	51.5 ± 20.3	**<0.001**
CKD III/IV/IV	392 (75.5)	167 (77.0)	559 (76.0)	0.678	191 (67.3)	74 (86.0)	265 (71.6)	**<0.001**
BB, N (%)	456 (87.0)	204 (92.7)	660 (88.7)	**0.025**	226 (78.5)	66 (76.7)	292 (78.1)	0.734
ACEI, N (%)	217 (41.4)	78 (35.5)	295 (39.7)	0.130	108 (37.5)	36 (41.9)	144 (38.5)	0.466
ARB, N (%)	159 (30.3)	52 (23.6)	211 (28.4)	0.064	76 (26.4)	33 (38.4)	109 (29.1)	0.042
ARNI, N (%)	68 (16.3)	46 (27.4)	114 (19.5)	**0.004**	11 (5.2)	3 (4.8)	114 (5.1)	0.912
MRA, N (%)	272 (51.9)	137 (62.3)	409 (55.0)	**0.010**	76 (26.4)	34 (39.5)	110 (29.4)	**0.019**
SGLT-2 inhibitors, N (%) N = 336	25 (10.7)	28 (27.2)	53 (15.8)	**<0.001**	3 (2.7)	5 (18.5)	9 (5.7)	**0.001**
Loop diuretics, N (%)	408 (77.9)	189 (85.9)	597 (80.2)	**0.012**	204 (70.8)	66 (76.7)	270 (72.2)	0.283
Statins, N (%)	337 (64.3)	181 (82.3)	518 (69.6)	**<0.001**	159 (55.2)	53 (61.6)	212 (56.7)	0.322
ASS, N (%)	170 (32.4)	103 (46.8)	273 (36.7)	**<0.001**	102 (35.4)	35 (40.7)	137 (36.6)	0.372
NOAC, N (%)	268 (51.1)	100 (45.5)	368 (49.5)	0.157	140 (48.6)	38 (44.2)	178 (47.6)	0.471
P2Y12 inhibitor, N (%)	118 (22.5)	72 (32.7)	190 (25.5)	**0.004**	55 (19.1)	19 (22.1)	74 (19.8)	0.541
**Echocardiography**								
LVEF (%)	39.3 ± 15.2	36.0 ± 12.8	38.4 ± 14.6	**0.008**	54.8 ± 13.5	53.2 ± 13.1	54.4 ± 13.4	0.377
LVEDd (mm) N = 807	61.1 ± 11.8	62.5 ± 11.3	62.5 ± 11.3	0.201	56.1 ± 10.3	56.9 ± 9.1	56.3 ± 10.03	0.569
LVESd (mm) N = 765	48.1 ± 13.9	49.7 ± 12.9	48.6 ± 13.6	0.223	37.6 ± 10.7	39.8 ± 10.3	38.1 ± 10.7	0.183
IVSd (mm) N = 830	10.7 ± 2.3	11.0 ± 8.0	10.8 ± 4.8	0.548	11.0 ± 2.3	11.5 ± 2.5	11.1 ± 2.3	0.184

LA diameter (mm) N = 851	54.9 ± 9.2	54.0 ± 7.7	54.6 ± 8.8	0.289	55.6 ± 10.1	54.4 ± 9.1	55.3 ± 9.9	0.437
mPG pre (mmHg)	1.6 ± 1.3	1.8 ± 1.3	1.7 ± 1.3	0.160	2.0 ± 1.2	2.2 ± 1.3	2.1 ± 1.2	0.297
mPG post (mmHg)	3.4 ± 1.6	3.4 ± 1.6	3.4 ± 1.6	0.745	3.8 ± 1.7	4.3 ± 1.8	3.9 ± 1.8	0.079
Grade of TR	1.9 ± 0.9	1.6 ± 1.0	1.8 ± 1.0	**<0.001**	1.7 ± 1.0	1.6 ± 1.1	1.7 ± 1.0	0.204
Severe TR (Grade III)	183 (34.9)	58 (26.4)	241 (32.4)	**0.023**	85 (29.5)	25 (29.1)	110 (29.4)	0.937
sPAP (mmHg)	45.1 ± 24.9	39.4 ± 26.5	43.4 ± 25.5	**0.006**	41.0 ± 26.9	42.6 ± 28.2	41.4 ± 27.2	0.617
**Procedural Outcomes**								
Grade of MR I-IV	3.6 ± 0.5	3.7 ± 0.5	3.6 ± 0.5	**0.038**	3.8 ± 0.4	3.9 ± 0.4	3.8 ± 0.4	0.620
III	204 (38.9)	68 (30.9)	272 (36.6)	**0.038**	47 (16.3)	16 (18.6)	63 (16.8)	0.619
IV	320 (61.1)	152 (69.1)	472 (63.4)		241 (83.7)	70 (41.4)	311 (83.2)	
Grade of MR post Clip	1.3 ± 0.7	1.2 ± 0.7	1.3 ± 0.7	0.785	1.4 ± 0.7	1.3 ± 0.8	1.4 ± 0.7	0.169
Grade ≤I	371 (70.8)	144 (65.5)	515 (69.2)	0.149	176 (61.1)	60 (69.8)	236 (63.1)	0.144
Procedure time (s) N = 862	5152.7 ± 3806.7	5405.2 ± 2300.0	5227.6 ± 3452.9	0.619	5222.3 ± 1981.6	5450.5 ± 3097.5	5267.5 ± 2224.1	0.691
Fluoroscopy time (s) N = 862	1613.2 ± 858.0	1666.0 ± 848.3	1628.3 ± 854.8	0.502	1798.1 ± 884.5	1595.6 ± 924.3	1754.3 ± 895.5	0.118
Number of implanted devices	1.5 ± 0.8	1.5 ± 0.7	1.5 ± 0.8	0.630	1.6 ± 1.0	1.4 ± 0.9	1.6 ± 0.92	0.061

Values are shown as frequencies (N) and percentages (%), mean ± standard deviation (SD).
Abbreviations: BMI = body mass index (kg/m²); CAD = coronary artery disease; MI = myocardial infarction; COPD = chronic obstructive pulmonary disease; AF = atrial fibrillation; LBBB = left bundle branch block; CRT = cardiac resynchronization therapy; DCM = dilatative cardiomyopathy; DMR = degenerative mitral regurgitation; FMR = functional mitral regurgitation; NYHA = New York Heart Association; STS = Society of Thoracic Surgeons; NT-proBNP = N-terminal pro hormone brain natriuretic peptide; eGFR = estimated glomerular filtration rate; BB = beta blocker; ACEI = angiotensin-converting enzyme inhibitor; ARB = AT receptor blocker; ARNI = angiotensin–neprilysin inhibitor; MRA = mineralocorticoid receptor antagonist; SGLT-2 = sodium-glucose cotransporter-2; ASS = acetylic salicylic acid ; NOAC = novel oral anticoagulant; P2Y12 inhibitor = adenosine diphosphate receptor antagonists; MR = mitral regurgitation; MV = mitral valve; LVEF = left-ventricular ejection fraction; LVEDd = left-ventricular end-diastolic diameter; LVESd = left-ventricular end-systolic diameter; LA = left atrium; IVSd = septum diameter; sPAP = systolic pulmonary artery pressure; TR = tricuspid regurgitation; PG = pressure gradient; MR = mitral regurgitation.

Supplemental Table S2. Patients (overall cohort) grouped according to the endpoint of death/rehospitalization for heart failure

Parameter	Endpoint not reached (N = 706)	Endpoint reached (N = 412)	Total (N = 1118)	p
Age	76.8 ± 9.1	77.8 ± 8.5	77.1 ± 8.6	0.065

BMI (kg/m²) N = 1113	26.1 ± 4.9	26.0 ± 4.7	26.0 ± 4.8	0.702
Sex, N (female) (%)	325 (46.0)	143 (34.7)	468 (41.9)	**<0.001**
Diabetes, N (%)	183 (25.9)	123 (29.9)	306 (27.4)	0.155
Arterial hypertension, N (%)	552 (78.2)	341 (82.8)	893 (79.9)	0.065
CAD, N (%)	448 (63.5)	291 (79.6)	739 (66.1)	**0.014**
Prior MI	147 (20.8)	128 (31.1)	275 (24.6)	**<0.001**
Hyperlipidemia, N (%)	402 (56.9)	250 (60.7)	652 (58.3)	0.221
Pulmonary hypertension, N (%)	275 (39.0)	155 (37.6)	430 (38.5)	0.659
COPD, N (%)	65 (9.2)	57 (13.8)	122 (10.9)	**0.017**
Smoker (current/former), N (%)	151 (21.4)	83 (20.1)	234 (20.9)	0.622
Family disposition, N (%)	95 (13.5)	68 (16.5)	163 (14.6)	0.163
AF, N (%)	448 (63.5)	280 (68.0)	728 (65.1)	0.127
LBBB, N (%) (N = 1111)	122 (17.3)	88 (21.4)	210 (18.8)	0.092
CRT-D/P, N (%)	50 (7.1)	45 (10.9)	95 (8.5)	**0.026**
DMR, N (%)	240 (34.0)	134 (32.5)	374 (33.5)	0.544
FMR, N (%)	335 (47.5)	209 (50.7)	544 (48.7)	
Mixed etiology, N (%)	131 (18.6)	69 (16.7)	200 (17.9)	
FMR/mixed, N (%)	466 (66.0)	278 (67.5)	744 (71.9)	0.615
DCM, N (%)	130 (18.4)	87 (21.8)	217 (19.4)	0.270
NYHA II, N (%)	131 (18.6)	34 (8.3)	165 (14.8)	**<0.001**
NYHA III, N (%)	433 (61.3)	226 (54.9)	659 (58.9)	
NYHA IV, N (%)	142 (20.1)	152 (36.9)	294 (26.3)	
EuroSCORE II	6.7 ± 6.1	10.8 ± 9.7	8.2 ± 7.9	**<0.001**
STS risk of mortality score	4.6 ± 4.3	6.6 ± 8.3	5.3 ± 6.2	**<0.001**
Troponin T pre (µg/L) N = 969	99.6 ± 714.3	100.8 ± 407.1	100.0 ± 625.1	0.978
NT-pro BNP pre (pg/mL) N = 933	4257.4 ± 5452.5	7124.3 ± 6896.2	5213.0 ± 6120.0	**<0.001**
Hemoglobin level (g/dL) N = 1111	12.6 ± 1.8	11.9 ± 2.0	12.3 ± 1.9	**<0.001**
eGFR (mL/min) N = 1106	51.6 ± 19.8	44.0 ± 19.5	48.8 ± 20.0	**<0.001**
CKD III/IV	487 (69.8)	337 (82.6)	824 (74.5)	**<0.001**
BB, N (%)	600 (85.0)	352 (85.4)	952 (85.2)	0.838
ACEI, N (%)	267 (37.8)	172 (41.7)	439 (39.3)	0.194
ARB, N (%)	209 (29.6)	111 (26.9)	320 (28.6)	0.342
ARNI, N (%)	89 (14.9)	39 (14.9)	128 (14.9)	0.982
MRA, N (%)	314 (44.5)	205 (49.8)	519 (46.4)	0.088
SGLT-2 inhibitors, N (%) N = 476	53 (13.9)	8 (8.5)	61 (12.8)	0.163
Loop diuretics, N (%)	524 (74.2)	343 (83.3)	867 (77.5)	**<0.001**
Statins, N (%)	465 (65.9)	265 (64.3)	730 (65.3)	0.603
ASS, N (%)	248 (35.1)	162 (39.3)	410 (36.7)	0.161
NOAC, N (%)	368 (52.1)	178 (43.2)	546 (48.8)	**0.004**
P2Y12 inhibitor, N (%)	170 (24.1)	94 (22.8)	264 (23.6)	0.631
**Echocardiography**				
LVEF (%)	45.0 ± 15.2	40.6 ± 17.1	43.4 ± 16.1	**<0.001**

LVEDd (mm) N = 807	58.7 ± 11.1	61.8 ± 11.8	59.8 ± 11.4	**<0.001**
LVESd (mm) N = 765	43.3 ± 13.1	48.2 ± 14.1	45.1 ± 13.6	**<0.001**
IVSd (mm) N = 830	11.1 ± 4.8	10.7 ± 2.5	10.9 ± 4.2	0.188
LA diameter (mm) N = 851	54.1 ± 9.0	56.2 ± 9.4	54.8 ± 9.2	**0.002**
mPG pre (mmHg)	1.8 ± 1.1	1.9 ± 1.7	1.8 ± 1.3	0.107
mPG post (mmHg)	3.5 ± 1.6	3.6 ± 1.9	3.6 ± 1.7	0.367
Grade of TR	1.8 ± . 1.0	2.0 ± 0.9	1.9 ± 1.0	0.084
Severe TR (Grade III)	204 (28.9)	147 (35.7)	351 (31.4%)	**0.018**
sPAP (mmHg)	42.8 ± 25.4	42.6 ± 27.2	42.7 ± 26.1	0.883
**Procedural Outcomes**				
Grade of MR I-IV	3.6 ± 0.5	3.8 ± 0.4	3.7 ± 0.5	**<0.001**
III	251 (35.6)	84 (20.4)	335 (30.0)	**<0.001**
IV	455 (64.4)	328 (79.6)	783 (70.0)	
Grade of MR post Clip	1.2 ± 0.7	1.4 ± 0.7	1.3 ± 0.7	**<0.001**
Grade ≤I	504 (71.4)	247 (60.0)	751 (67.2)	**<0.001**
Procedure time (s) N = 315	5251.8 ± 3274.6	5167.5 ± 1808.2	5239.8 ± 3105.7	0.866
Fluoroscopy time (s) N = 580	1632.5 ± 860.7	1730.4 ± 882.6	1669.5 ± 869.8	0.109
Number of implanted devices	1.6 ± 0.9	1.4 ± 0.8	1.5 ± 0.9	**0.020**
Cardiogenic shock, N (%)	5 (0.7)	17 (4.1)	22 (2.0)	**<0.001**
Infection	25 (3.5)	43 (10.4)	68 (6.1)	**<0.001**
Pneumonia	13 (1.8)	27 (6.6)	40 (3.6)	**<0.001**
Sepsis	1 (0.1)	16 (3.9)	17 (1.5)	**<0.001**

Values are shown as frequencies (N) and percentages (%), mean ± standard deviation (SD).
Abbreviations: BMI = body mass index (kg/m^2); CAD = coronary artery disease; MI = myocardial infarction; COPD = chronic obstructive pulmonary disease; AF = atrial fibrillation; LBBB = left bundle branch block; CRT = cardiac resynchronization therapy; DCM = dilatative cardiomyopathy; DMR = degenerative mitral regurgitation; FMR = functional mitral regurgitation; NYHA = New York Heart Association; STS = Society of Thoracic Surgeons; NT-proBNP = N-terminal pro hormone brain natriuretic peptide; eGFR = estimated glomerular filtration rate; BB = beta blocker; ACEI = angiotensin-converting enzyme inhibitor; ARB = AT receptor blocker; ARNI = angiotensin–neprilysin inhibitor; MRA = mineralocorticoid receptor antagonist; SGLT-2 = sodium-glucose cotransporter-2; ASS = acetylic salicylic acid ; NOAC = novel oral anticoagulant; P2Y12 inhibitor = adenosine diphosphate receptor antagonists; MR = mitral regurgitation; MV = mitral valve; LVEF = left-ventricular ejection fraction; LVEDd = left-ventricular end-diastolic diameter; LVESd = left-ventricular end-systolic diameter; LA = left atrium; IVSd = septum diameter; sPAP = systolic pulmonary artery pressure; TR = tricuspid regurgitation; PG = pressure gradient; MR = mitral regurgitation.

Supplemental Table S3. Patients (DMR only) grouped according to the endpoint of death/rehospitalization for heart failure				
**Parameter**	**Endpoint not reached (N = 240)**	**Endpoint reached (N = 134)**	**Total (N = 374)**	**p**
Age	78.5 ± 8.3	80.1 ± 6.7	79.0 ± 7.8	0.055
BMI (kg/m²)	25.9 ± 4.5	25.9 ± 5.1	25.9 ± 4.7	0.976
Sex, N (female) (%)	122 (50.8)	53 (39.6)	175 (46.8)	**0.036**
Diabetes, N (%)	44 (18.3)	42 (31.3)	86 (23.0)	**0.004**
Arterial hypertension, N (%)	188 (78.3)	111 (82.8)	299 (79.9)	0.297
CAD, N (%)	130 (54.2)	88 (65.7)	218 (58.3)	**0.030**
Prior MI	29 (12.1)	23 (17.2)	52 (13.9)	0.17
Hyperlipidemia, N (%)	122 (50.8)	71 (53.0)	193 (51.6)	0.690
Pulmonary hypertension, N (%)	88 (36.7)	59 (44.0)	147 (39.3)	0.162
COPD, N (%)	20 (8.3)	18 (13.4)	38 (20.2)	0.118
Smoker (current/former), N (%)	49 (20.4)	19 (14.2)	68 (18.2)	0.134
Family disposition, N (%)	23 (9.6)	19 (14.2)	42 (11.2)	0.177
AF, N (%)	149 (62.1)	92 (68.7)	241 (64.4)	0.203
LBBB, N (%) (N=1111)	23 (9.6)	16 (11.9)	39 (10.4)	0.475
CRT-D/P, N (%)	5 (2.1)	3 (2.2)	8 (2.1)	1.0
DCM, N (%)	20 (8.3)	10 (7.5)	30 (8.0)	0.766
NYHA II, N (%)	50 (20.8)	6 (4.5)	56 (15.0)	**<0.001**
NYHA III, N (%)	140 (58.3)	86 (64.2)	226 (60.4)	
NYHA IV, N (%)	50 (20.8)	42 (31.3)	92 (24.6)	
EuroSCORE II	6.3 ± 6.9	9.4 ± 9.1	7.4 ± 7.9	**<0.001**
STS risk of mortality score	4.3 ± 4.3	7.1 ± 9.9	5.3 ± 7.0	**<0.001**
Troponin T pre (µg/L) N=311	30.6 ± 32.4	45.7 ± 39.5	35.6 ± 35.6	**<0.001**
NT-pro BNP pre (pg/mL) N=303	3076.9 ± 4298.0	5418.3 ± 6252.0	3834.2 ± 5122.1	**<0.001**
Hemoglobin level (g/dL) N=371	12.6 ± 1.7	11.9 ± 2.1	12.4 ± 1.9	**<0.001**
eGFR (mL/min)	55.1 ± 19.4	45.1 ± 20.3	51.5 ± 20.3	**<0.001**
CKD III/IV	155 (65.7)	110 (82.1)	265 (71.6)	**<0.001**
BB, N (%)	189 (78.8)	103 (76.9)	292 (78.1)	0.673
ACEI, N (%)	88 (36.7)	56 (41.8)	144 (38.5)	0.329
ARB, N (%)	75 (31.3)	34 (25.4)	109 (29.1)	0.230
ARNI, N (%)	5 (2.6)	9 (10.7)	14 (5.1)	**0.013**
MRA, N (%)	62 (25.8)	48 (35.8)	110 (29.4)	0.042
SGLT-2 inhibitors, N (%) N=140	6 (5.2)	2 (8.0)	8 (5.7)	0.587
Loop diuretics, N (%)	161 (67.1)	109 (81.3)	270 (72.2)	**0.003**
Statins, N (%)	146 (60.8)	66 (49.3)	212 (56.7)	**0.030**
ASS, N (%)	95 (39.6)	42 (31.3)	137 (36.6)	0.113
NOAC, N (%)	119 (49.6)	59 (44.0)	178 (47.6)	0.302
P2Y12 inhibitor, N (%)	44 (18.3)	30 (22.4)	74 (19.8)	0.345
**Echocardiography**				

LVEF (%)	54.8 ± 12.4	53.8 ± 15.2	54.4 ± 13.4	0.515
LVEDd (mm)<br>N=261	56.1 ± 10.2	56.6 ± 9.8	56.3 ± 10.0	0.737
LVESd (mm)<br>N=251	37.5 ± 10.4	39.2 ± 11.1	38.1 ± 10.7	0.222
IVSd (mm)<br>N=268	11.1 ± 2.2	11.2 ± 2.6	11.1 ± 2.3	0.687
LA diameter (mm)<br>N=274	54.3 ± 9.0	57.4 ± 11.2	55.3 ± 9.9	**0.016**
mPG pre (mmHg)	2.1 ± 1.3	2.1 ± 1.2	2.1 ± 1.2	0.897
mPG post (mmHg)	3.7 ± 1.6	4.3 ± 1.9	3.9 ± 1.8	**0.016**
Grade of TR	1.7 ± 1.0	1.7 ± 1.1	1.7 ± 1.0	0.65
Severe TR (Grade III)	67 (27.9)	43 (32.1)	110 (29.4)	0.396
sPAP (mmHg)	41.9 ± 25.8	40.3 ± 29.6	41.3 ± 27.2	0.577
**Procedural Outcomes**				
Grade of MR I-IV	3.8 ± 0.4	3.9 ± 0.3	3.8 ± 0.4	**<0.001**
III	53 (22.1)	10 (7.5)	63 (16.8)	**<0.001**
IV	171 (87.7)	82 (88.2)	253 (87.8)	
Grade of MR post Clip	1.3 ± 0.7	1.5 ± 0.8	1.4 ± 0.7	0.054
Grade ≤I	161 (67.1)	75 (56.0)	236 (63.1)	**0.003**
Procedure time (s)<br>N=96	5306.0 ± 2249.2	5021.5 ± 2125.0	5267.5 ± 2224.1	0.670
Fluoroscopy time (s)<br>N=580	1757.0 ± 947.4	1749.3 ± 795.1	1754.3 ± 895.5	0.945
Number of implanted devices<br>N=344	1.6 ± 1.0	1.5 ± 0.9	1.6 ± 0.9	0.501
Cardiogenic shock, N (%)	1 (0.4)	7 (5.2)	8 (2.1)	**0.002**
Infection	12 (5.0)	11 (8.2)	23 (6.2)	0.219
Pneumonia	7 (2.9)	5 (3.7)	12 (3.2)	0.673
Sepsis	1 (0.4)	5 (3.7)	6 (1.6)	**0.015**

Values are shown as frequencies (N) and percentages (%), mean ± standard deviation (SD).
Abbreviations: DMR = degenerative mitral regurgitation; BMI = body mass index (kg/m^2); CAD = coronary artery disease; MI = myocardial infarction; COPD = chronic obstructive pulmonary disease; AF = atrial fibrillation; LBBB = left bundle branch block; CRT = cardiac resynchronization therapy; DCM = dilatative cardiomyopathy; NYHA = New York Heart Association; STS = Society of Thoracic Surgeons; NT-proBNP = N-terminal pro hormone brain natriuretic peptide; eGFR = estimated glomerular filtration rate; BB = beta blocker; ACEI = angiotensin-converting enzyme inhibitor; ARB = AT receptor blocker; ARNI = angiotensin–neprilysin inhibitor; MRA = mineralocorticoid receptor antagonist; SGLT-2 = sodium-glucose cotransporter-2; ASS = acetylic salicylic acid ; NOAC = novel oral anticoagulant; P2Y12 inhibitor = adenosine diphosphate receptor antagonists; MR = mitral regurgitation; MV = mitral valve; LVEF = left-ventricular ejection fraction; LVEDd = left-ventricular end-diastolic diameter; LVESd = left-ventricular end-systolic diameter; LA = left atrium; IVSd = septum diameter; sPAP = systolic pulmonary artery pressure; TR = tricuspid regurgitation; PG = pressure gradient; MR = mitral regurgitation.

Supplemental Table S4. Diabetics grouped according to the endpoint of death/rehospitalization for heart failure				
**Parameter**	**Endpoint not reached (N = 183)**	**Endpoint reached (N = 123)**	**Total (N=306)**	**p**
Age	76.1 ± 8.3	75.9 ± 8.1	76.0 ± 8.2	0.885
BMI (kg/m^2)	27.6 ± 5.1	27.8 ± 5.2	27.7 ± 5.2	0.683
Sex, N (female) (%)	81 (44.3)	43 (35.0)	124 (40.5)	0.104
Arterial hypertension, N (%)	156 (85.2)	113 (91.9)	269 (87.9)	0.081
CAD, N (%)	132 (72.1)	98 (79.7)	230 (75.2)	0.134
Prior MI	57 (31.1)	51 (41.5)	108 (35.3)	0.064
Hyperlipidemia, N (%)	113 (61.7)	83 (67.5)	196 (64.1)	0.306
Pulmonary hypertension, N (%)	70 (38.3)	39 (31.7)	109 (35.6)	0.241
COPD, N (%)	23 (12.6)	17 (13.8)	40 (13.1)	0.750
Smoker (current/former), N (%)	47 (25.7)	32 (26.0)	79 (25.8)	0.948
Family disposition, N (%)	22 (12.0)	28 (22.8)	50 (16.3)	**0.013**
AF, N (%)	114 (62.3)	72 (58.5)	186 (60.8)	0.509
LBBB, N (%) (N=1111)	36 (19.7)	34 (27.6)	236 (77.1)	0.104
CRT-D/P, N (%)	14 (7.7)	14 (11.4)	28 (9.2)	0.267
DMR, N (%)	44 (24.0)	42 (34.1)	86 (28.1)	0.087
FMR, N (%)	106 (57.9)	67 (54.5)	173 (56.5)	
Mixed etiology, N (%)	33 (18.0)	14 (11.4)	47 (15.3)	
FMR/mixed, N (%)	139 (76.0)	81 (65.9)	220 (71.9)	0.054
DCM, N (%)	31 (16.9)	22 (17.9)	53 (17.3)	0.830
NYHA II, N (%)	21 (11.5)	18 (14.6)	39 (12.7)	0.09
NYHA III, N (%)	114 (62.3)	61 (49.6)	157 (57.2)	
NYHA IV, N (%)	48 (26.2)	44 (35.8)	92 (30.1)	
EuroSCORE II N=306	7.8 ± 6.4	11.1 ± 9.3	9.1 ± 7.8	**<0.001**
STS risk of mortality score N=303	5.3 ± 4.3	116.0 ± 1215.4	50.2 ± 774.4	0.057
Troponin T pre (µg/L) N=256	47.7 ± 92.5	162.1 ± 671.1	91.9 ± 426.0	**0.036**
NT-pro BNP pre (pg/mL) N=252	4991.6 ± 5894.4	7010.8 ± 7009.5	5712.7 ± 6375.6	**0.016**
Hemoglobin level (g/dL) N=304	12.2 ± 1.9	11.6 ± 2.1	12.0 ± 2.1	**0.015**
eGFR (mL/min) N=303	47.6 ± 19.7	42.8 ± 18.7	45.7 ± 19.4	**0.035**
CKD III/IV/IV	137 (75.3)	104 (86.0)	241 (79.5)	**0.024**
BB, N (%)	164 (89.6)	106 (86.2)	270 (88.2)	0.360
ACEI, N (%)	67 (36.6)	47 (38.2)	114 (37.3)	0.777
ARB, N (%)	47 (25.7)	38 (30.9)	85 (27.8)	0.318
ARNI, N (%) N=230	34 (21.5)	15 (20.8)	49 (21.3)	0.906
MRA, N (%)	102 (55.7)	69 (56.1)	171 (55.9)	0.950
SGLT-2 inhibitors, N (%)	28 (15.3)	5 (4.1)	33 (10.8)	**<0.001**
Loop diuretics, N (%)	148 (80.9)	107 (87.0)	255 (83.3)	0.159

Statins, N (%)	145 (79.2)	89 (72.4)	234 (76.5)	0.164
ASS, N (%)	75 (41.0)	63 (51.2)	138 (45.1)	0.078
NOAC, N (%)	92 (50.3)	46 (37.4)	138 (45.1)	**0.026**
P2Y12 inhibitor, N (%)	52 (28.4)	39 (31.7)	91 (29.7)	0.537
**Echocardiography**				
LVEF (%)	42.7 ± 14.4	37.5 ± 15.5	40.7 ± 15.0	**0.005**
LVEDd (mm) N=218	59.7 ± 10.4	63.2 ± 11.8	61.0 ± 11.0	**0.024**
LVESd (mm) N=209	44.8 ± 12.2	50.4 ± 13.6	47.0 ± 13.0	**0.003**
IVSd (mm) N=226	11.3 ± 8.5	10.8 ± 2.9	11.1 ± 7.1	0.556
LA diameter (mm) N=226	53.9 ± 8.8	54.4 ± 6.7	54.1 ± 8.0	0.651
mPG pre (mmHg)	1.9 ± 1.2	2.0 ± 1.6	1.9 ± 1.3	0.556
mPG post (mmHg)	3.6 ± 1.6	3.8 ± 1.8	3.7 ± 1.7	0.579
Grade of TR	1.6 ± 1.0	1.6 ± 1.0	1.6 ± 1.0	0.618
Severe TR (Grade III)	49 (26.8)	34 (27.6)	83 (27.1)	0.867
sPAP (mmHg)	42.0 ± 26.5	37.9 ± 27.6	40.3 ± 27.0	0.198
**Procedural Outcomes**				
Grade of MR I-IV	3.7 ± 0.5	3.8 ± 0.4	3.7 ± 0.4	**0.043**
III	58 (31.7)	26 (21.1)	84 (27.5)	**0.042**
IV	125 (68.3)	97 (78.9)	222 (72.5)	
Grade of MR post Clip	1.2 ± 0.7	1.3 ± 0.6	1.3 ± 0.7	0.492
Grade ≤I	122 (66.7)	82 (66.7)	204 (66.7)	1.0
Procedure time (s) N=84	5426.8 ± 2603.5	5370.5 ± 1966.3	5415 ± 2477.1	0.934
Fluoroscopy time (s) N=227	1665.1 ± 930.2	1621.5 ± 775.3	1647.1 ± 867.9	0.710
Number of implanted devices	1.4 ± 0.5	1.3 ± 0.5	1.4 ± 0.5	0.164
Cardiogenic shock, N (%)	1 (0.5)	2 (1.6)	3 (1.0)	0.350
Infection, N (%)	5 (2.7)	10 (8.1)	15 (4.9)	**0.032**
Pneumonia, N (%)	2 (1.1)	6 (4.9)	8 (2.6)	**0.042**
Sepsis, N (%)	0	2 (1.6)	2 (0.7)	0.161

Values are shown as frequencies (N) and percentages (%), mean ± standard deviation (SD).

Abbreviations: BMI = body mass index (kg/m^2); CAD = coronary artery disease; MI = myocardial infarction; COPD = chronic obstructive pulmonary disease; AF = atrial fibrillation; LBBB = left bundle branch block; CRT = cardiac resynchronization therapy; DCM = dilatative cardiomyopathy; DMR = degenerative mitral regurgitation; FMR = functional mitral regurgitation; NYHA = New York Heart Association; STS = Society of Thoracic Surgeons; NT-proBNP = N-terminal pro hormone brain natriuretic peptide; eGFR = estimated glomerular filtration rate; BB = beta blocker; ACEI = angiotensin-converting enzyme inhibitor; ARB = AT receptor blocker; ARNI = angiotensin–neprilysin inhibitor; MRA = mineralocorticoid receptor antagonist; SGLT-2 = sodium-glucose cotransporter-2; ASS = acetylic salicylic acid ; NOAC = novel oral anticoagulant; P2Y12 inhibitor = adenosine diphosphate receptor antagonists; MR = mitral regurgitation; MV = mitral valve; LVEF = left-ventricular ejection fraction; LVEDd = left-ventricular end-diastolic diameter; LVESd = left-ventricular end-systolic diameter; LA = left atrium; IVSd = septum diameter; sPAP = systolic pulmonary artery pressure; TR = tricuspid regurgitation; PG = pressure gradient; MR = mitral regurgitation.
